# Ortner syndrome secondary to saccular thoracic aneurysm

**DOI:** 10.1016/j.jvscit.2021.05.005

**Published:** 2021-05-21

**Authors:** Brian M. Leoce, Jack T. Bernik, Brett Voigt, Herbert Dardik, Thomas R. Bernik

**Affiliations:** Department of Vascular Surgery, Englewood Hospital and Medical Center, Englewood, NJ

**Keywords:** Carotid–carotid bypass, Ortner syndrome, Thoracic aneurysm, Vocal cord paralysis

## Abstract

Mechanical stretching of the left laryngeal nerve secondary to an enlarged left atrium was first described by Dr Norbert Ortner in 1987. An extensive literature search revealed only 76 reported cases of Ortner syndrome, with the more recent reports describing other causes of the syndrome such as pulmonary hypertension, aortic dissection, and a thoracic aneurysm. We recently encountered this rare pathologic entity in an elderly man who had presented with severe hoarseness, presumed to be due to one of the aforementioned vascular anomalies. In the present report, we have highlighted the pathology and hybrid repair of this challenging entity.

## Case report

**Fig 1:**
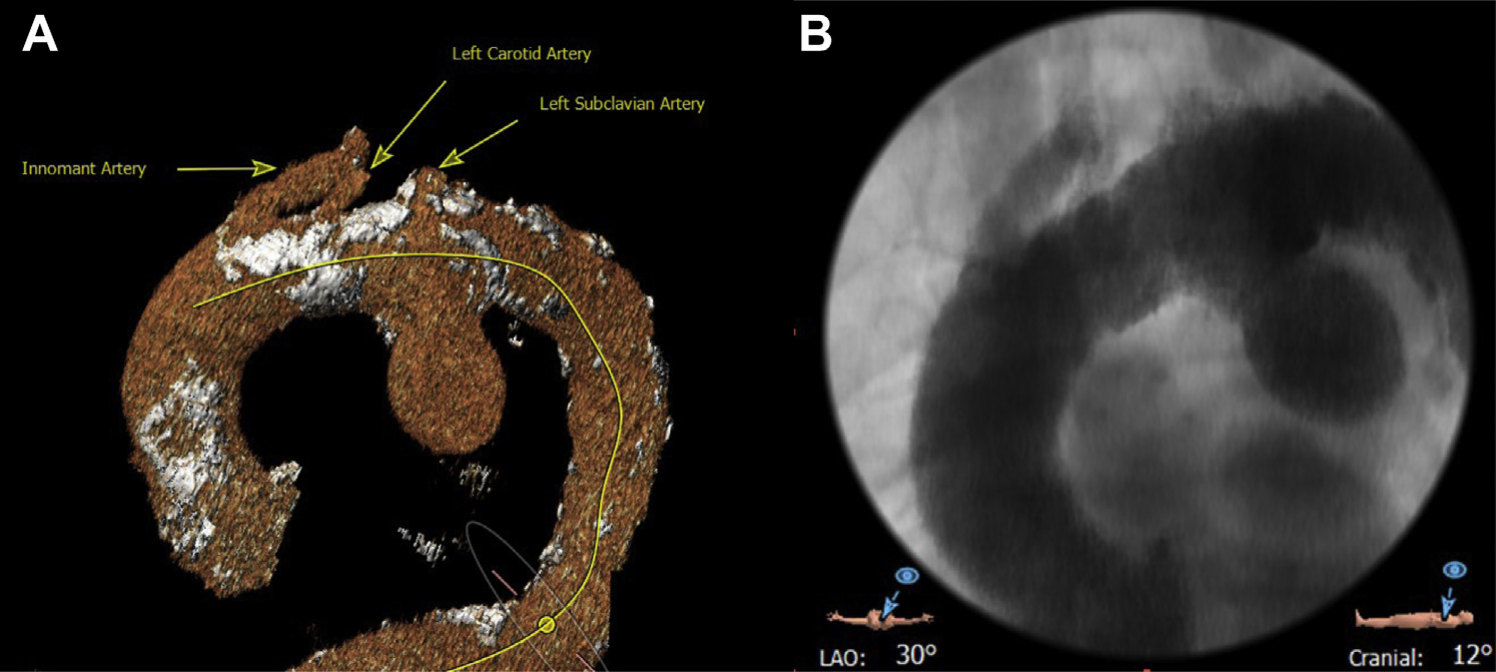
**A,** Computed tomography three-dimensional reconstruction showing a saccular thoracic aneurysm in relation to the aortic arch vessel anatomy. **B,** Intraoperative angiogram showing saccular thoracic aneurysm filling with contrast.

An 88-year-old man had presented with a sudden onset of severe hoarseness of his voice. The more common causes for his symptoms had been ruled out by pulmonology medicine and video laryngoscopy, which showed left vocal cord paralysis, before our examination. The patient's surgical history included uneventful staged bilateral carotid endarterectomy in 2002. Thoracic computed tomography revealed a 4.3 × 5.2 × 5.0 cm saccular thoracic aneurysm in zones 1 through 3 of his aorta (Fig 1, *A* and *B*). Because of the patient's age and multiple comorbidities, including calcific plaque in and near the ostium of all three brachiocephalic vessels, open debranching was not considered; rather, a staged hybrid repair was selected.

The initial procedure was a right-to-left carotid–carotid bypass performed via a retroesophageal route using a 6-mm ringed polytetrafluoroethylene graft. This was followed by ligation of the proximal left common carotid artery. Scarring from the patient's previous cervical interventions and calcification of the proximal common carotid arteries added to the technical challenge of the procedure. A left carotid–left subclavian bypass was not performed in the same setting because of further scarring at the left supraclavicular area; thus, revascularization of the left arm was planned for the endovascular portion in the subsequent procedure. Postoperatively, the patient showed no signs of a neurologic deficit apart from hoarseness that continued from his preoperative condition.

**Fig 2:**
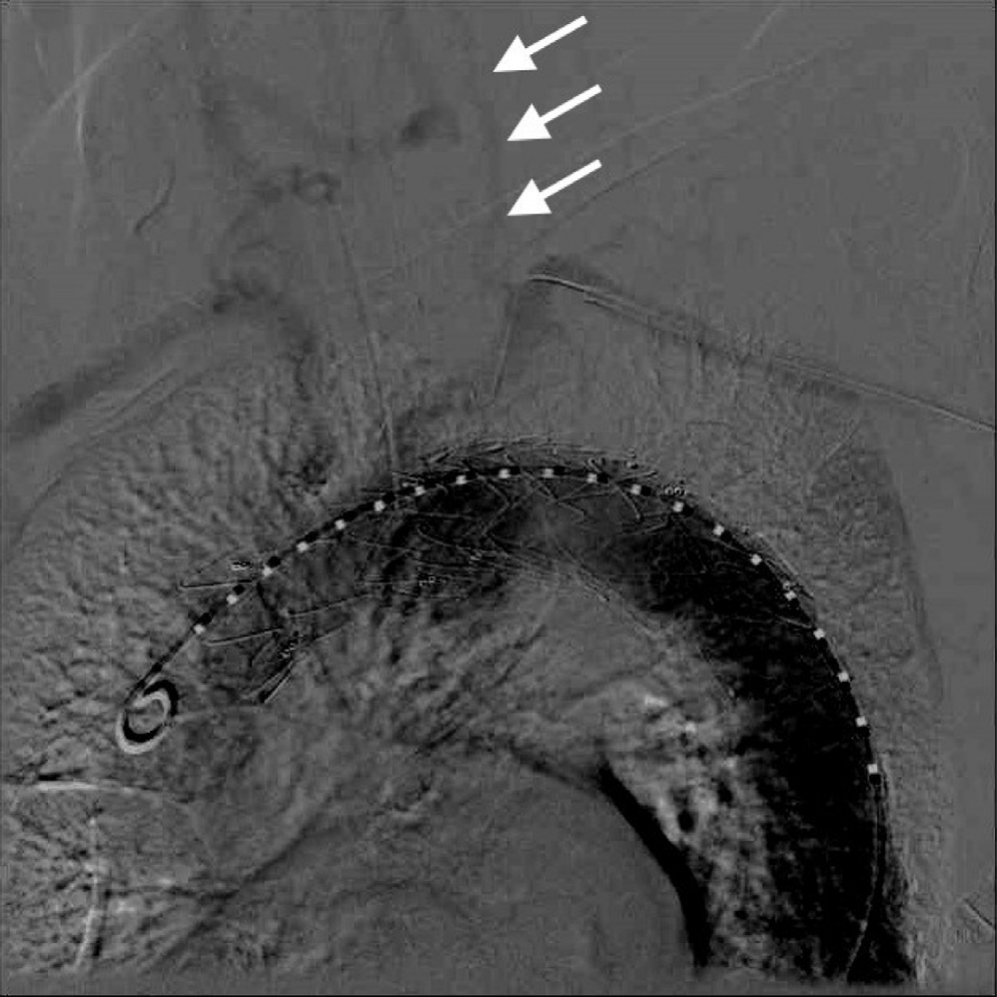
Intraoperative angiogram after endovascular covered graft placement demonstrating a type 2 endoleak via retrograde flow through the left vertebral artery (*arrows*) and subsequent perfusion of the left upper extremity.

The second procedure was initiated by direct cutdown and cannulation of the right femoral artery and left brachial artery, percutaneous access to the left femoral artery, and right radial artery access for placement of a marker wire in the brachiocephalic artery. A 38-mm covered stent-graft was deployed just before the origin of the innominate artery to cover the origin of the left carotid and left subclavian arteries. A second 34-mm covered stent-graft was placed distally. Type Ia and type II endoleaks were observed with initial angiography, the latter originating from retrograde flow via the left vertebral artery (Fig 2). The proximal landing zone of the covered stent-grafts was ballooned again, successfully eliminating the type Ia endoleak. Laser-directed graft fenestration at the left subclavian artery could not be performed because perpendicular alignment with the graft could not be achieved. The procedure was, therefore, terminated with a type II endoleak, which significantly decreased after heparin reversal. Embolization and extra-anatomic bypass would be considered, if required, if left upper extremity symptoms or a persistent leak developed. The patient was extubated without new-onset peripheral or neurologic ischemic symptoms.

**Fig 3:**
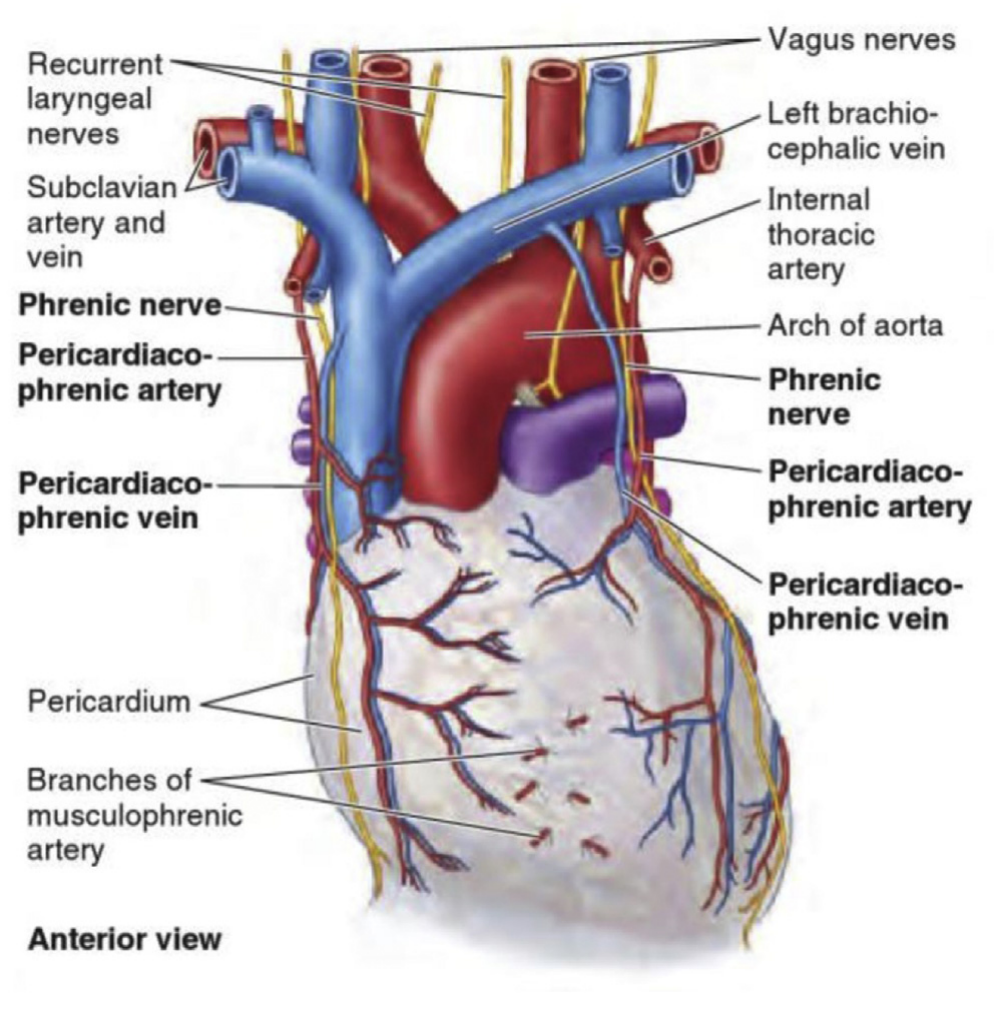
Artist's depiction of left recurrent laryngeal location and surrounding anatomy.

At the 2-week follow-up visit, satisfactory perfusion of the left upper extremity was present, with a nonpalpable left radial pulse, the trunk of the left subclavian artery was thrombosed, and left vertebral flow remained, as expected, reversed. For educational and anatomic reference, an artist's depiction of the left recurrent laryngeal nerve location and surrounding anatomy is shown in Fig 3.^1^ The patient provided written informed consent for the report of his case and imaging studies.

## Discussion

Ortner syndrome is a rare, left, recurrent laryngeal nerve palsy secondary to mechanical stretching by cardiovascular disease, usually an enlarged left atrium.^2^^,^^3^ Since its discovery, other causes of Ortner syndrome have been reported, including pulmonary hypertension, aortic dissection, and thoracic aneurysms.^4^, ^5^, ^6^ Ortner syndrome secondary to thoracic aneurysm compression has only been reported 24 times.

Several operative approaches are available for the treatment of zone 1 and zone 2 thoracic aortic arch aneurysms. However, our patient's age, previous carotid endarterectomies, and multiple comorbidities, including severe calcification of the aortic arch vessels, precluded open thoracic repair and traditional debranching. Parallel graft techniques are often used for inline flow to the left carotid and left subclavian arteries; however, the degree of calcification of the arch and limited endograft landing zones in the patient yielded too high a risk for repair of a type Ia endoleak. Approved fenestrated thoracic grafts are not readily available; however, physician-modified stent grafts have been used with a reasonable degree of success reported.^7^ Therefore, we chose a two-stage hybrid repair consisting of bypass, followed by endovascular aneurysm exclusion and, if needed, fenestration and embolization. In our experience, attempts to preserve left subclavian flow are warranted; however, in certain instances, in which the left subclavian origin is free from aneurysmal degeneration and stent-graft opposition is achieved, natural thrombosis can occur, obviating the need for embolization and possible left carotid–left subclavian artery bypass.

Multiple off-label techniques for in situ fenestration of aortic grafts and revascularization have been described, including laser-directed graft fenestration. Redlinger et al^8^ described 22 patients in whom they successfully cannulated the left subclavian via laser fenestration. In our patient, the ostium of the left subclavian artery was angled anteriorly, which inhibited our ability to properly align the laser perpendicular to the aortic stent-graft. Fenestration was, therefore, aborted. Left subclavian artery occlusion with short-segment thoracic stent-grafts has been well tolerated owing to the extensive collateral circulation, resulting in a low risk of spinal ischemia or posterior cerebral stroke.^9^ Embolization of the type II endoleak was not required because postoperative thrombosis had occurred. Retrograde vertebral flow and collateral flow prevented left arm ischemia. Therefore, extra-anatomic left arm revascularization (femoral–axillary or axillary–axillary bypass) was not required.

At 1 month after the procedure, the patient was doing well. His voice had improved mildly, and he had no ischemic arm symptoms. Duplex ultrasound confirmed a patent carotid–carotid bypass, ligation of the proximal common left carotid artery, thrombosis of the proximal left subclavian artery, and left vertebral artery flow reversal. Ultrasound also documented thrombosis of the saccular aneurysm. Postoperative computed tomography was not performed because of the patient's stage IV chronic kidney disease. The patient's left arm was pulseless; however, Doppler flow was audible, and his hand was fully functional. No symptoms of subclavian steal were present. The patient's hoarseness persisted, which was as expected because the aneurysm sac had been left in place.

Although a few cases of Ortner syndrome secondary to a thoracic aneurysm have been reported, the increasing life expectancy and an aging population are likely causative of the syndrome's increasing prevalence. When other more common causes of hoarseness of the voice have been excluded, a thoracic aortic aneurysm should be considered as a possible cause. Ortner syndrome secondary to mechanical compression by a thoracic aneurysm is typically coupled with advanced age and multiple comorbidities, and thus, a patient with an anatomically high risk of surgical repair. Because all cases will involve zone 1 and zone 2 arch pathology, we advocate a staged minimally invasive approach consisting of bypass, followed by stent-graft exclusion, and, if necessary, left subclavian revascularization.
